# Electroacupuncture Protects Cognition by Regulating Tau Phosphorylation and Glucose Metabolism via the AKT/GSK3β Signaling Pathway in Alzheimer’s Disease Model Mice

**DOI:** 10.3389/fnins.2020.585476

**Published:** 2020-11-20

**Authors:** Anping Xu, Qingtao Zeng, Yinshan Tang, Xin Wang, Xiaochen Yuan, You Zhou, Zhigang Li

**Affiliations:** ^1^School of Acupuncture-moxibustion and Tuina, Beijing University of Chinese Medicine, Beijing, China; ^2^Information Engineering Institute, Beijing Institute of Graphic Communication, Beijing, China; ^3^Department of Rehabilitation and Traditional Chinese Medicine, The Second Affiliated Hospital of Zhejiang University School of Medicine, Hangzhou, China; ^4^Beijing Hospital of Traditional Chinese Medicine, Capital Medical University, Beijing, China; ^5^Key Laboratory of Microcirculation, Ministry of Health, Institute of Microcirculation, Chinese Academy of Medical Sciences, Peking Union Medical College, Beijing, China

**Keywords:** Alzheimer’s disease, electroacupucnture, cognition, glucose metabolism, tau, AKT/GSK3β pathway

## Abstract

**Background:**

Alzheimer’s disease (AD) is mainly manifested as a continuous and progressive decline in cognitive ability. Neurofibrillary tangles (NFTs) are pathological hallmarks of AD and due to accumulated phosphorylated Tau. Glycogen synthase kinase-3β (GSK3β), as a major Tau kinase and a downstream target of the serine protein kinase B (AKT) signaling pathway, can regulate Tau phosphorylation in AD. Importantly, the AKT/GSK3β signaling pathway is involved in glucose metabolism, and abnormal glucose metabolism is found in the AD brain. Numerous studies have shown that electroacupuncture (EA), which is thought to be a potential complementary therapeutic approach for AD, can protect cognitive ability to a certain extent.

**Objective:**

The purpose of this experiment was to investigate whether the protective and beneficial mechanism of EA on cognition was mediated by the AKT/GSK3β signaling pathway, thereby improving glucose metabolism and Tau phosphorylation in the brain.

**Methods:**

EA was applied to the Baihui (GV20) and Yintang (GV29) acupoints of 6-month-old amyloid precursor protein (APP)/presenilin-1 (PS1) mice for 20 min, and then quickly prick Shuigou (GV26) acupoint. The intervention was performed once every other day for 28 days. The Morris water maze (MWM) test was performed on C57BL/6N (Non-Tg) mice, APP/PS1 (Tg) mice and EA-treated Tg (Tg + EA) mice to evaluate the effect of EA therapy on cognitive function. ^18^F-FDG positron emission tomography (PET), immunohistochemistry, and western blotting (WB) were used to investigate the possible mechanism underlying the effect of EA on AD.

**Results:**

EA treatment significantly improved the cognition of APP/PS1 mice and the glucose uptake rate in the hippocampus. Furthermore, EA inhibited the phosphorylation of Tau (Ser199 and Ser202) proteins by inducing AKT (Ser473) and GSK3β (Ser9) phosphorylation.

**Conclusion:**

These results demonstrate that EA intervention protects cognition by enhancing glucose metabolism and inhibiting abnormal phosphorylation of Tau protein in the AD model mice, and the AKT/GSK3β pathway might play an irreplaceable role in the regulation process.

## Introduction

According to the World Alzheimer Report 2018, there is one new case of dementia every 3 s across the world. Fifty million people worldwide were living with dementia in 2018, but this number will more than triple to 152 million by 2050. A systematic analysis of the global burden of disease found that dementia was the fastest-growing cause of death between 2005 and 2015, increasing by 40% (GBD 2015 Mortality and Causes of Death Collaborators, 2016). The main clinical manifestations of Alzheimer’s disease (AD) are progressive episodic memory disorder, cognitive dysfunction, and decreased ability in activities of daily living. However, treating AD remains challenging. There are currently two types of drugs available, cholinesterase inhibitors and NMDA receptor antagonists, both of which aim to treat some of the symptoms of AD but cannot prevent the progression of the disease (Alzheimer Disease Agents, 2012). AD has become a growing public health problem, and effective treatments are still lacking.

Pathologically, AD is characterized by amyloid plaques and neurofibrillary tangles (NFTs), the main components of which are amyloid-β (Aβ) peptide and Tau protein, respectively, in the brain (Guillozet et al., 2003). Previously, the development of AD therapy mainly focused on Aβ, while Tau has attracted more attention in recent years because of the neurotoxicity of its hyperphosphorylated form (Giacobini and Gold, 2013; Slomski, 2018). Glycogen synthase kinase-3β (GSK3β) is a major Tau kinase. Substantial evidence has shown that GSK3β, which functions as a downstream target of AKT, can regulate both Tau phosphorylation and Aβ production in AD through the PI3K/AKT-dependent pathway (Hernandez et al., 2010). Also, neural activity and function are highly dependent on the continuous supply of glucose. Decreased intake of glucose may be the direct substrate of cognitive impairment in AD (Kuehn, 2020). Interestingly, glucose metabolism is closely related to the activation of AKT/GSK3β pathway, especially involved in the phosphorylation of AKT and GSK3β (Griffith et al., 2019). The above indicated that abnormal changes in phosphorylated Tau and glucose metabolism in AD are closely associated with the AKT/GSK3β pathway (Tokutake et al., 2012).

Acupuncture is a unique therapy used to treat diseases in China. Under the guidance of traditional Chinese medicine (TCM) theory, acupuncture needles are inserted into the body at a certain angle, and acupuncture techniques such as twisting, lifting, and thrusting are used to stimulate specific parts of the body to treat a disease. The insertion point is called the acupuncture point. Electroacupuncture (EA) involves stimulation by a pulsating electrical current through acupuncture needles. In animal and clinical trials, EA have shown unique protective effects in inhibiting neuronal apoptosis and neuroinflammation as well as in promoting cognitive function (Wang et al., 2012; Su et al., 2019). At the same time, Our previous experiments have confirmed that EA could alleviate cognitive impairment by promoting glucose metabolism in the brain of mice (Xu et al., 2020).

As the understanding of AD has advanced, increasing evidences have shown that the mechanism of AD is very complex with the further study of AD (Yu et al., 2018). In the past, we studied the effects of EA on Tau and glucose metabolism separately but neglected to explore the connection between its effects on these processes. Based on the abovementioned findings, the study investigated whether the cognitive protective effect of EA in regulating Tau protein and glucose metabolism is associated with the activation of the AKT/GSK3β signaling pathway. As a good animal model of AD, the amyloid precursor protein (APP)/presenilin-1 (PS1) mouse strain exhibits the pathological characteristics of AD patients to some extent. Therefore, we first assessed changes in the cognitive abilities of these mice with the Morris water maze (MWM) test. To demonstrate that EA can improve the cognitive abilities of mice, the possible mechanism underlying the effect of EA was further studied. Subsequently, we observed glucose metabolism in the brain of mice after EA intervention using ^18^F-FDG positron emission tomography (PET). Finally, we detected Tau, AKT, GSK3β and their phosphorylation using immunohistochemical staining and western blotting (WB), aiming to elucidate the protective mechanism of EA on cognition.

## Materials and Methods

### Experimental Animals

**FIGURE 1:**
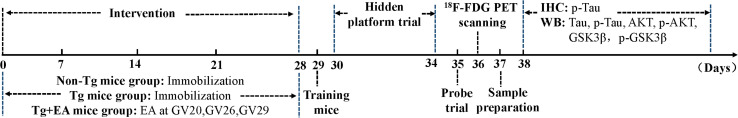
The timeline of the study exploring the effect of EA on the cognition of APP/PS1 mice.

Amyloid precursor protein/presenilin-1 mice, which overexpress the human APP and PS1 mutations, were obtained from Beijing HFK Bioscience Co., Ltd. [experimental animal license number: SCXK (Jing) 2014-0004]. The mice were housed one per cage in an environment with a temperature of 23 ± 2°C and humidity of 50 ± 10% under a 12-h light/dark cycle (lights on 08:00–20:00 h). *Ad libitum* access to water and food was provided. 6-month-old male APP/PS1 transgenic mice were randomly assigned to Tg group or Tg + EA group, with ten mice per group. Age-matched C57BL/6N (Non-Tg) mice were used as controls. After 7 days of acclimation, the mice began to receive EA treatment. All experimental procedures were carried out in strict accordance with the regulations of the National Institutes of Health guide for the care and use of laboratory animals. The timeline of the experimental design is shown in Figure 1.

### EA Intervention

Based on our previous studies, the acupoint prescription included GV20, GV29, and GV26. According to the Acupoint Standard for Experimental Animals, GV20 is at the middle point of the parietal bone of mice, GV26 is located 1 mm below the tip of the nose of mice, and GV29 is located in the depression between the eyes of mice. The Tg + EA mice were treated with EA. Firstly, we immobilize the mice with special bags based on the size of the mice. Then, one needle was inserted in a backward direction at GV20, and the other needle was inserted toward the tip of the nose at GV29. The insertion depth at the two acupoints was 5 mm. After the needle handles were fixed, the two needles were connected to the EA device. Parameters were set to 2 Hz and 1 mA. After 20 min, turn off the EA apparatus and a quick prick was delivered at GV26. Acupuncture points were stimulated with disposable sterile needles (0.25 mm × 13 mm). The mice in the Non-Tg and Tg groups were immobilized in mouse bags only. The interventions described above were administered once every other day for 28 days.

### MWM Test

The MWM test is a classic experiment that assesses cognitive abilities by analyzing rodent behavior. The hidden platform trial of the MWM can be performed to analyze the spatial learning ability, and the probe trial can be used to assess spatial memory ability of mice (Vorhees and Williams, 2006). On day 29 of this study, all the mice were trained to swim in the pool. The hidden platform trials were performed after 24 h (Skelton et al., 2007). A circular platform was placed in a fixed position in the southwest (SW) quadrant of the pool. The mice were placed into the water with their heads facing the pool wall. The experiment was performed 4 times, with each mouse starting from each of the four quadrants. The interval between trials was 20 min, and the training trials were performed for 5 days. The time it took the mice to find the underwater platform (escape latency) was recorded, with the maximum latency being 60 s. The probe trial was performed on day 35. The mice were placed into pool (without the circular platform) in the northeast (NE) quadrant, and their performance was recorded for 60 s. The number of times each mouse passed the platform was recorded, and the time each mouse spent in the platform quadrant and swimming trajectories were analyzed (Dong et al., 2015).

### ^18^F-FDG PET

On day 36, ^18^F-FDG PET imaging was conducted. First, the blood glucose levels of the mice were measured to ensure that the values were 7.0–10.0 mmol/L. Then, the mice were banned from drinking water for 6 h before anesthesia. After completely anesthetized, the mice were injected with 14.8–16.5 MBq ^18^F-FDG in the tail vein. Waiting for 60 min, micro-PET images were collected for 10 min. Single frame micro-PET images were capture, and then image reconstruction was carried out. The following steps were manually selection the hippocampus regions of interest (ROIs) from transverse, coronal, and sagittal PET/CT images by the experimenter and the uptake rate of ^18^F-FDG per gram were analyzed (Xu et al., 2020).

### Immunohistochemistry

The ABC method of immunohistochemistry was performed. Paraffin slices were dewaxed. 0.1 mol/L citrate buffer for antigen repair for 10 min. The serum of 5% normal sheep were sealed at 37°C for 30 min. A primary antibody against p-Tau (Ser199) (1:600) was added to the tissue and incubated at 4°C overnight. The following day the slices were rinsed with PBS for 3 times, and then the secondary antibody was added and incubated for 10 min. After the slices were rinsed with PBS for 3 times, AB compound were added and incubated for 90 min. After being rinsed again with PBS, the slices were colored, redyed, dehydrated, and made transparent and sealed. The brain slices were observed with a microscope. The information of the primary antibody is listed in Table 1.

**TABLE 1:** The information of the primary antibodies used in this experiment.

**Antibody**	**Antibody Code**	**Company**
Anti-Tau antibody	ab75714	Abcam, England
Anti-Tau (phospho S199) antibody	ab4749	Abcam, England
Anti-Tau (phospho S202) antibody	ab108387	Abcam, England
Anti-pan-AKT antibody	ab8805	Abcam, England
Anti-AKT1 (phospho S473) antibody	ab8932	Abcam, England
Anti-GSK3 beta antibody	ab93926	Abcam, England
Anti-GSK3 beta (phospho S9) antibody	ab131097	Abcam, England
Anti-GAPDH antibody	ab8245	Abcam, England

### Western Blotting

The mice hippocampal tissues were quickly stripped after they were sacrificed. The total proteins were extracted from the tissue and the protein concentration were measured by BCA method. After detecting the protein content of the sample, extracted proteins were separated by 10% SDS-PAGE. The voltage of SDS-PAGE electrophoresis separation glue and concentrated glue was 120 and 80 V, respectively. After the proteins were transferred onto PVDF membranes at 200 mA, rinsed the membranes and sealed them at 4°C overnight. The primary antibodies against Tau (1:2000), p-Tau (Ser199) (1:500), p-Tau (Ser202) (1:5000), AKT (1:300), p-AKT (Ser473) (1:200), GSK3β (1:800), and p-GSK3β (Ser9) (1:500) primary antibodies were added and incubated at 4°C overnight. The secondary antibody (Shanghai, Jiehao, Haopoly-HRP, 1:5000) were added and incubated at room temperature for 1 h. After rinsed, ECL luminescent solution was added, and the cassette was exposed. An antibody against GAPDH (1: 2000, TA-08, Zibo, China) was used as an internal control. The information of the primary antibodies are listed in Table 1.

### Statistical Analysis

SPSS 20.0 statistical software was used for data analysis. Data were presented as means ± SD. Multivariate analysis of variance (ANOVA) with repeated measurement design data was used to analyze the difference of escape latency in each group of mice. For the remaining data except escape latency, if the data were normally distributed and had homogeneous variance, one-way analysis of variance was used and LSD test was used for inter-group comparison. If the data were abnormally distributed or the variance was uneven, non-parametric test was used. Statistical significance was set at *P* < 0.05, and high statistical significance was set at *P* < 0.01.

## Results

### Effect of EA on the Cognitive Abilities of APP/PS1 Mice

**FIGURE 2:**
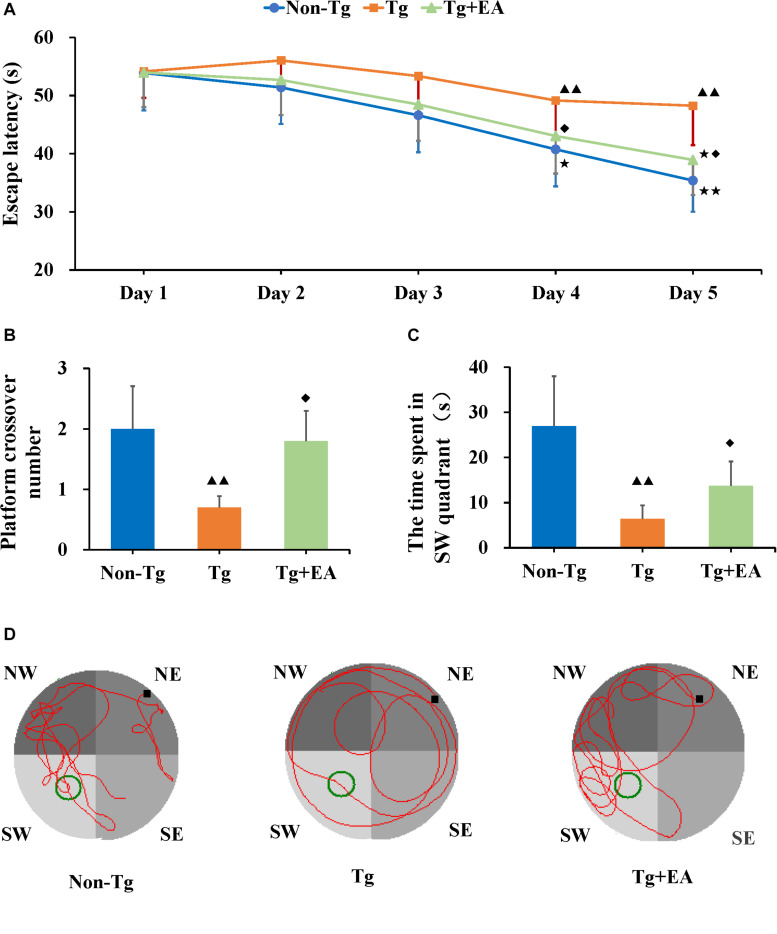
The Results of MWM test. **(A)** The change trend of escape latencies, and comparison of escape latencies between each group. **(B,C)** Comparison of the number of platform crossing and the time spent in the SW quadrant by each group. **(D)** Representative swimming trajectories of mice from each group. *n* = 10, means ± SD. ^★★^*P* < 0.01, ^★^*P* < 0.05 compared with day 1. ^▲▲^*P* < 0.01 compared with the Non-Tg group. ^◆^*P* < 0.05 compared with the Tg group.

In the hidden platform trial of the MWM, the spatial learning abilities of APP/PS1 mice were assessed from day 30 to 34. The escape latency was measured as the time it took a mouse to find the hidden platform fixed in position underwater. The escape latency of the Tg group was maintained at a high level, but that escape latencies of the Non-Tg and Tg + EA groups showed an obvious downward trend over the training phase (Figure 2A). A shorter escape latency across training days indicates better learning ability. The results suggested that the mice from the Tg group exhibited learning disabilities. However, compared with Tg group, the escape latency of Tg + EA group decreased gradually and was significantly shortened on days 4 and 5. (Figure 2A), suggesting that EA had a protective effect on the learning abilities of APP/PS1 mice.

On day 35, the platform was removed from the southwest (SW) quadrant, and the probe trial was conducted to evaluate the maintenance of memory (Thong-asa et al., 2013). Compared to the Non-Tg, the Tg group exhibited notably fewer platform crossings and spent markedly less time in the SW quadrant. The shorter time spent by the APP/PS1 mice in the SW quadrant, which had been the location of the platform, implies that they exhibited worse memory (Tian et al., 2019). The Tg + EA group stayed obviously longer in the SW quadrant than the Tg group (Figures 2B,C). The results suggested that EA treatment significantly promoted memory retention in APP/PS1 mice. Furthermore, we analyzed the swimming trajectories of the mice. It was found that the trajectories of the Tg group were random, whereas the mice from the Non-Tg group and Tg + EA group exhibited trajectories that were mostly concentrated in the SW and northwest (NW) quadrants and passed the platform position several times (Figure 2D). Based on the MWM results, EA therapy was beneficial to the cognitive performance of AD model mice, which was related to the protection of spatial learning and memory ability.

### Effect of EA on Glucose Metabolism in the Hippocampi of APP/PS1 Mice

**FIGURE 3:**
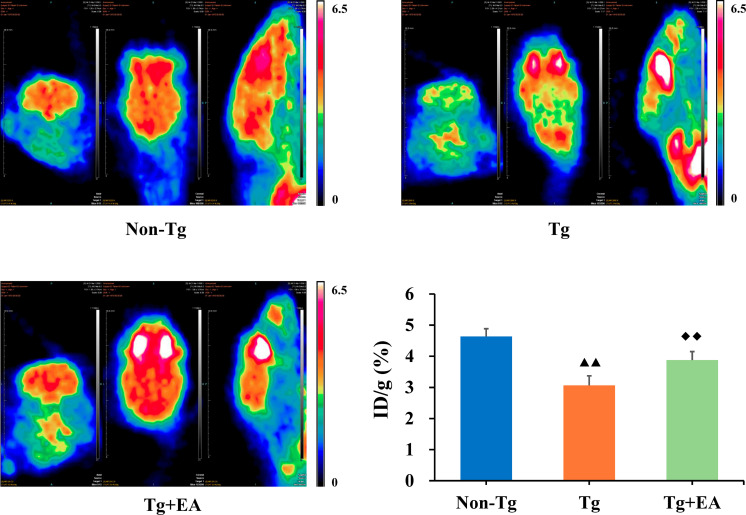
^18^F-FDG PET imaging and uptake rate in the hippocampi of APP/PS1 mice. *n* = 4. Data are presented as means ± SD. Color code: min = 0, max = 6.5. ^▲▲^*P* < 0.01 compared with the Non-Tg group. ^◆◆^*P* < 0.01 compared with the Tg group.

Dysregulation of glucose metabolism in the brain are a key sign of the development of AD (Mosconi, 2005). To assess glucose metabolism, ^18^F-FDG PET was performed after the MWM test. We selected the ROIs in the hippocampus on PET images and further analyzed the glucose metabolism rate by calculating the uptake rate of ^18^F-FDG per gram in the hippocampus of each group. PET imaging showed that the glucose metabolism rate of the Tg group was lower than the glucose metabolism rates of the Non-Tg and Tg + EA groups. Furthermore, the data obtained from PET imaging confirmed that the uptake rate of ^18^F-FDG in the hippocampus of the Tg group were obviously lower than that in the Non-Tg group but that significantly increased after EA intervention (Figure 3).

### Effect of EA on the Expression of Tau in the Hippocampi of APP/PS1 Mice

**FIGURE 4:**
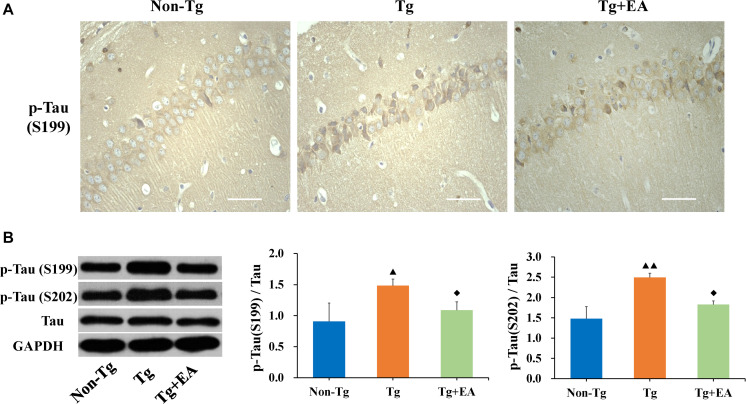
Comparison of the expression of Tau in the hippocampi of APP/PS1 mice. **(A)** Representative immunohistochemistry images of p-Tau (Ser199) in the hippocampus in each group. The scale bar is 50 μm. **(B)** The relative expressions of hippocampal phosphorylated Tau and total Tau and the ratios of p-Tau (Ser199 and Ser202) expression level to the total Tau level in APP/PS1 mice by WB analysis. *n* = 6, means ± SD. ^▲^*P* < 0.05, ^▲▲^*P* < 0.01 compared with the Non-Tg group. ^◆^*P* < 0.05 compared with the Tg group.

The formation of NFTs by hyperphosphorylated Tau is considered a crucial event in the pathogenesis of AD (Alonso et al., 1994). We hypothesized that the improvement of cognition by EA is related to the regulation of abnormal Tau phosphorylation. Therefore, we next evaluated the expression of Tau, including phosphorylated Tau and total Tau, in the hippocampus. As expected, we observed an obvious increase in the localization of p-Tau (Ser199) immunopositivity in the hippocampus in the Tg group compared to the Non-Tg group and found that the optical density of p-Tau (Ser199) was significantly higher in the Tg group than the Non-Tg and Tg + EA groups (Figure 4A). WB results confirmed that p-Tau (Ser199 and Ser202) levels were obviously increased in the hippocampus in the Tg group compared with the Non-Tg group and were decreased after EA in the Tg + EA group (Figure 4B). In brief, the results of WB and immunohistochemistry showed that the neuroprotection of EA was achieved with modulating Tau hyperphosphorylation.

### Effect of EA on the AKT/GSK3β Signaling Pathway in the Hippocampi of APP/PS1 Mice

**FIGURE 5:**
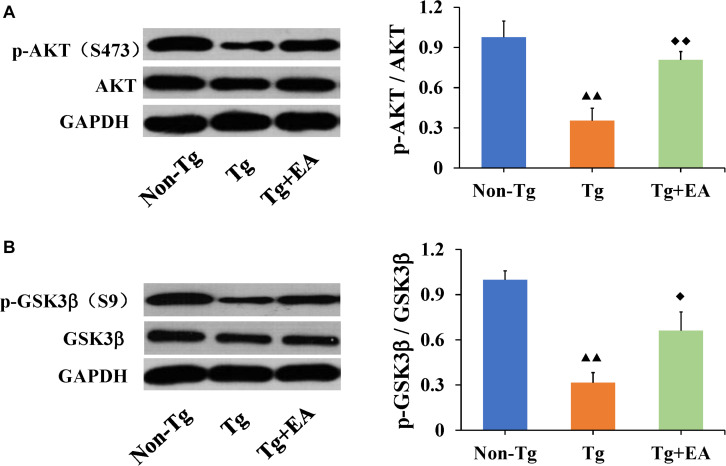
Comparison of the expressions of AKT and GSK3β in the hippocampi of APP/PS1 mice. **(A)** The relative expressions of hippocampal p-AKT (Ser473) and total AKT, and the ratio of p-AKT (Ser473) expression level to the total AKT level in APP/PS1 mice by WB analysis. **(B)** The relative expressions of hippocampal p-GSK3β (Ser9) and total GSK3β, and the ratio of p-GSK3β (Ser9) expression level to the total GSK3β level in APP/PS1 mice by WB analysis. *n* = 6, means ± SD. ^▲▲^*P* < 0.01 compared with the Non-Tg group. ^◆◆^*P* < 0.01, ^◆^*P* < 0.05 compared with the Tg group.

Glycogen synthase kinase-3β is one of the most important kinases for abnormal phosphorylation of Tau protein and is one of the major downstream substrates of AKT (Hernandez et al., 2013). Meanwhile, the phosphorylation of AKT and GSK3β are regulated by integrated signals derived from glucose. Activation of AKT results in a substantial increase in p-AKT (Ser473), which leads to increased phosphorylation of its downstream substrate GSK3β (Ser9), thereby reducing GSK3β activity. We speculated that the effect of EA on glucose metabolism and Tau was related to the AKT/GSK3β pathway. To test this possibility, we next examined the activation of the AKT/GSK3β signaling pathway. The results showed that EA had no significant differences in the total protein expressions of AKT or GSK3β in the hippocampus among groups. However, the levels of both p-AKT (Ser473) and p-GSK3β (Ser9) were significantly increased in the Tg + EA group compared to the Tg group (Figure 5). Taken together, these findings suggested that EA treatment activated the AKT/GSK3β signaling pathway of APP/PS1 mice. Activation of AKT results in a substantial increase in p-AKT (Ser473), which leads to increased phosphorylation of its downstream substrate GSK3β (Ser9), thereby reducing GSK3β activity.

## Discussion

In this study, we investigated the mechanism by which EA intervention affects cognition in the APP/PS1 mouse strain, a rodent model of AD. We found that EA therapy was beneficial in improving cognitive decline by promoting glucose uptake in the hippocampus and that the underlying molecular mechanisms might be associated with phosphorylation of Tau protein through the AKT/GSK3β signaling pathway.

Individuals can experience gradual progressive cognitive decline that results from AD pathology in the brain. When cognitive impairment becomes sufficient to interfere with daily function, the patient is diagnosed with AD (Albert et al., 2011). Cognitive decline is a major issue that affects the quality of life of AD patients (Zhao et al., 2020). Clinical studies have shown that acupuncture reduces cognitive impairment in AD patients (Feng et al., 2012; Tan et al., 2017). In addition, studies have shown that acupuncture is indeed effective in improving cognitive function in AD animal models compared with the placebo effect. According to the theory of TCM, meridian blockage in the brain is the pathological basis of AD and aggravates the abnormal brain function. The governor vessel (GV) is closely related to the central nervous system, and the acupoints on GV are the first choice for the treatment of central nervous system related diseases. In most studies, GV20 is chosen as the main treatment acupoint, and it has been proven to be effective in the treatment of AD mice with EA (Lin et al., 2016). According to the traditional Chinese classics, we proposed a new acupuncture therapy, a “dredge GV and refresh mind therapy,” in which EA at GV20, GV26, and GV29 can improve brain function and prevent cognitive decline in AD (Deng et al., 2016; Tang et al., 2019).

Learning and memory are higher neural activities and higher functions of the brain. The MWM test is a classical and the most common method for evaluating memory and learning changes in AD mice. In the MWM test, which assesses spatial learning, the rodents use signs on the walls of the circular swimming pool to find an underwater platform from different falling points (Zhao et al., 2014). Spatial learning ability was assessed by the results of swimming training, and spatial memory level was assessed by analyzing the preference of the mice to the location of the platform after the platform was removed (Neufeld et al., 2019). The MWM has proven to be a reliable test to assess cognitive ability of the rodents, and the behavior of animals in experiments is strongly correlated with hippocampal synaptic plasticity and NMDA receptor function (Pinho et al., 2017). Our results showed that from day 2, the escape latency of the Non-Tg group showed a downward trend, while that of the Tg group did not change, indicating that the learning ability of the Tg mice was significantly impaired. In addition, the mice in the Tg group crossed the platform fewer times and spent less time in the SW quadrant than the mice in the Non-Tg group. Spatial reference memory refers to the ability to complete a spatial positioning task through multiple learning and is an important measure in learning and memory research and for nervous system function assessment. The results suggested that the Tg mice exhibited memory impairments, which is in accordance with what is observed following AD-related pathological changes. Importantly, the effect of EA treatment from day 1 to 3 showed a good trend. Compared with that of the Tg group, the cognitive ability of the Tg + EA group was significantly improved on days 4 and 5, suggesting that EA had an obvious effect on AD. Based on the “treating pre-disease” theory of TCM, we hypothesized that earlier EA intervention might have a better effect on protecting cognition. Providing intervention before cognitive impairment develops may be more beneficial (Ding et al., 2020).

Moreover, in adult humans, the brain uses approximately 20% of the energy in the body. Glucose consumption is tightly linked to neuronal activity and neuronal function. Regional metabolic aberrations underlie the functional and cognitive decline seen in patients with AD (Shen et al., 2019; Kuehn, 2020). PET scans provide functional information that is unique and cannot be obtained using other types of imaging. Hence, ^18^F-FDG PET, which offers acceptable sensitivity and accuracy, is recognized as a potential tool for pre-symptomatic diagnosis of AD (Shen et al., 2019; Blazhenets et al., 2020). In AD, numerous interrelations between abnormal glucose metabolism and the occurrence of brain lesions have been described. First, AD could be a partial consequence of insulin resistance, which affects insulin signaling and favors abnormal deposition of Aβ and phosphorylated Tau accumulation in the brain, leading to cognitive decline (Malkki, 2015). Abnormalities of glucose metabolism occur in the early stages of AD, involving the temporal and parietal lobes. In animal models, patients and people at high risk of AD showed this characteristic (Arrieta-Cruz and Gutierrez-Juarez, 2016). Using ^18^F-FDG PET, we observed that EA treatment activated the hippocampus, suggesting that EA enhanced glucose metabolism and contributed to energy metabolism, thus improving the cognitive function of the Tg mice (De Santi et al., 2001).

Increasing evidence suggests that Tau protein is one of the most peculiar proteins in the central nervous system (de Calignon et al., 2012). The highly flexible structure of Tau protein allows interactions with multiple partners, suggesting that Tau is involved in numerous signaling pathways. The accumulation of Tau, especially hyperphosphorylated Tau, which is a major component of neurofibrillary lesions characteristic of AD and other brain disorders (Karikari et al., 2020), is more compatible with the clinical severity and progression of pathological findings in AD than Aβ (Rapoport et al., 2002). Studies on the correlation between cognitive impairment and histopathological changes have consistently demonstrated that the number of NFTs, not the number of plaques, correlates best with the presence and/or degree of dementia in AD (Bittar et al., 2020). The neurodegenerative synaptic dysfunction is associated with the abnormal expression Tau. The more phosphorylated Tau deposition in the brain of AD patients, the lower their cognitive score (Hoover et al., 2010). Meanwhile, FDG PET show that pathological Tau is consistent with regions with low glucose metabolism in the brain (Baghel et al., 2019). Tau protein phosphorylation mainly occurs in serine or threonine residues, and there are many phosphorylation sites. We chose Ser199 and Ser202 as the representative phosphorylation sites. Phosphorylation of a number of serine phosphorylation sites of Tau, including Ser199, is elevated in AD mice (Neddens et al., 2018). Our results showed that phosphorylation of Tau at Ser199 and Ser202 were significantly increased in the hippocampus in the Tg group compared to the Non-Tg group, but that the level of total Tau was not significantly changed. This suggested that acupuncture has an effect against AD by efficiently inhibiting Tau phosphorylation.

Glycogen synthase kinase-3β is a serine/threonine-protein kinase that is essential for energy metabolism and nerve cell development (Hooper et al., 2008). Moreover, GSK3β promotes actin and tubulin assembly, processes required for synaptic reorganization during memory formation, which is critical for the induction of memory formation, switching off LTD, and allowing LTP to occur (Peineau et al., 2007). Besides, our previous study found that EA therapy can improve the expression of NMDARs in hippocampus, and EA may regulate the LTP mediated by NMDARs, enhance cognitive ability. Substantial evidence has revealed that GSK3β, which functions as a major Tau kinase and a downstream target of the PI3K/AKT signaling pathway, regulates both Tau phosphorylation and Aβ production in AD (Qi et al., 2017). In our study, the levels of p-AKT and p-GSK3β in the hippocampus in the Tg + EA group were dramatically increased compared with those in the Tg group, but the total expression of AKT and GSK3β were unaffected. Phosphorylation increased AKT activity but decreased GSK3β activity. Our results suggested that EA activated AKT to promote the phosphorylation of GSK3β (Ser9), by reducing GSK3β activity, and ultimately inhibit the phosphorylation of Tau in the hippocampus, thereby protecting cognitive function. It is important to note that the fluctuations in blood glucose levels can affect the phosphorylation of AKT and GSK3β. In other words, the activities of AKT and GSK3β are regulated by integrated signals from glucose. Conversely, inhibition of GSK3β can also regulate glucose levels in animal models of insulin resistance. In view of this, GSK3β has attracted increasing attention as a potential therapeutic target in the treatment of diabetes (Maqbool and Hoda, 2017). As an integrator of cellular glucose sensors and multiple signals, activation of the AKT/GSK3β signaling pathway may affect neurondysfunction resulting from changes in glucose availability, such AD (Clodfelder-Miller et al., 2005). Besides, GSK3β can inhibit the activity of glycogen synthesis and reduce the synthesis of glycogen *in vivo*. On the other hand, GSK3β can indirectly inhibit the synthesis of glycogen by affecting insulin signaling pathway. Therefore, we speculate that the mechanism by which EA regulates brain glycometabolism might partly involve the AKT/GSK3β signaling pathway. More importantly, GSK3β is the intersection of Tau phosphorylation and glucose metabolism abnormalities in AD.

**FIGURE 6:**
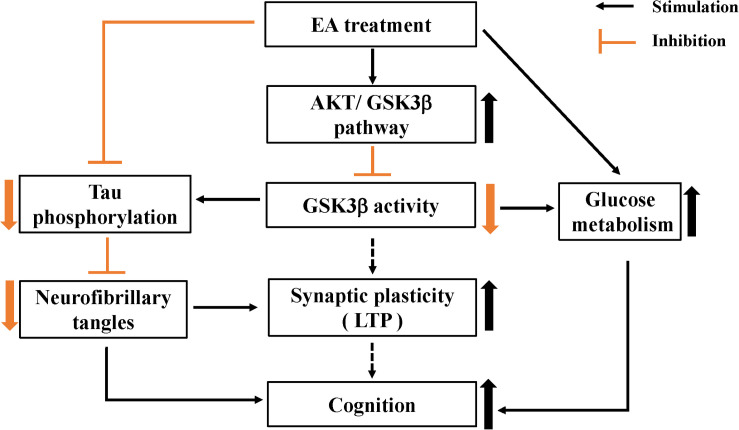
Schematic representation of EA protective effects on cognition in APP/PS1 mice.

In summary, this study provides evidence for the protective effect of EA intervention on cognition, with EA tending to be beneficial for enhancing learning and memory abilities in AD model mice. In addition, this study reveals the mechanism underlying the protective effect of EA on cognition. We found that EA has an effect on the AKT/GSK3β signaling pathway, as reflected by increased phosphorylation of AKT and GSK3β, and that a reduction in GSK3β activity contributes to improvements in glucose metabolism and inhibition of abnormal Tau phosphorylation (Figure 6). The mechanisms underlying the protective effect of EA on cognition could involve multiple processes. The multitarget effect of acupuncture is appropriate given the complexity of AD pathogenesis. Future studies clarifying the mechanism underlying the effect of EA in AD should be encouraged.

## Data Availability Statement

The data analyzed in this study is subject to the following licenses/restrictions: The raw data supporting the conclusions of this article will be made available by the authors, without undue reservation, to any qualified researcher. Requests to access these datasets should be directed to Anping Xu, xuanping01@163.com.

## Ethics Statement

The animal study was reviewed and approved by the Medicine and Animal Ethics Committee of the Beijing University of Chinese Medicine.

## Author Contributions

AX: experimental design, data analysis, and manuscript preparation. QZ and YT: experimental design and manuscript preparation. XW and XY: data collection. YZ and ZL: experimental design. All authors contributed to draft the manuscript and have read and approved the final manuscript.

## Conflict of Interest

The authors declare that the research was conducted in the absence of any commercial or financial relationships that could be construed as a potential conflict of interest.
